# Multidisciplinary Cancer Management of Colorectal Cancer in Tikur Anbessa Specialized Hospital, Ethiopia

**DOI:** 10.1200/JGO.19.00014

**Published:** 2019-10-07

**Authors:** Biniyam Tefera Deressa, Nikola Cihoric, Ephrem Tefesse, Mathewos Assefa, Daniel Zemenfes

**Affiliations:** ^1^ Addis Ababa University, Addis Ababa, Ethiopia; ^2^ Bern University Hospital, University of Bern, Bern, Switzerland

## Abstract

**PURPOSE:**

Multidisciplinary cancer care is currently considered worldwide as standard for the management of patients with cancer. It improves patient diagnostic and staging accuracy and provides patients the benefit of having physicians of various specialties participating in their treatment plan. The purpose of this study was to describe the profile of patients discussed in the Tikur Anbessa Multidisciplinary Tumor Board (MTB) and the potential benefits brought by multidisciplinary care.

**METHODS:**

The study involved the retrospective assessment of all patient cases presented to the Tikur Anbessa Hospital colorectal cancers MTB between March 2016 and November 2017. The data were collected from the MTB medical summary documents and were analyzed using SPSS version 20 (SPSS, Chicago, IL).

**RESULTS:**

Of 147 patients with colorectal cancer, 96 (65%) were men. The median age at presentation was 46 years (range, 17-78 years). The predominant cancer was rectal (n = 101; 69%), followed by colon (n = 24; 16%). Of these, 68 (45%) and 22 (15%) had stage III and IV disease, respectively, on presentation to the MTB. The oncology department presented the majority of the patients for discussion. Most patients had undergone surgery before the MTB discussion but had no proper preoperative clinical staging information. The majority of patients with rectal cancer treated before the MTB discussion had undergone surgery upfront; however, most of the patients who were treatment naive before MTB received neoadjuvant chemoradiotherapy before surgery.

**CONCLUSION:**

Decisions made by tumor boards are more likely to conform to evidence-based guidelines than are those made by individual clinicians. Therefore, early referral of patients to MTB before any treatment should be encouraged. Finally, other hospitals in Ethiopia should take a lesson from the Tikur Anbessa Hospital colorectal cancers MTB and adopt multidisciplinary cancer management.

## INTRODUCTION

Cancer continues to be a medical challenge worldwide. It has become one of the leading causes of morbidity and mortality, particularly in low- and middle-income countries, where 60% of the world's total new patient cases are diagnosed.^1^ Cancer is a complex disease that is rarely detected, diagnosed, and adequately treated by a single physician or discipline; hence, a multidisciplinary approach is required.^2^ Multidisciplinary care can be broadly defined as an integrated team approach to health care in which medical and allied health care professionals consider all relevant treatment options and collaboratively develop an individual treatment plan for each patient.^3^ Multidisciplinary care can be delivered either by specialized units or by multidisciplinary tumor boards (MTBs).^2,4^ The initial evaluation and treatment decisions are the most critical to the outcome of a patient, and multidisciplinary care provides patients with cancer the potential benefit of having physicians of various specialties participate in their treatment planning.^5^

There is evidence that multidisciplinary cancer care improves patient diagnostic accuracy, staging accuracy, and overall survival.^6-10^ This is why multidisciplinary cancer care is currently considered worldwide as standard for the management of patients with cancer. Increasingly, multidisciplinary care is practiced through MTBs or multidisciplinary teams (MDT) meetings, which are formal meetings usually held regularly, often on a weekly basis.^4^ MTB or MDT meetings bring together physicians from different disciplines (oncologists, radiologists, surgeons, pathologists) and various members of the health care team who are involved in a patient’s care, for inter- and intradisciplinary discussions. These discussions help in planning the necessary diagnostics or treatment strategy for individual patients on the basis of the available scientific evidence and resources, with the aim of standardizing and improving outcomes.^11^

As with the management of other cancers, managing colorectal cancer through a single treatment is difficult. Because treatment options for colorectal cancer have increased rapidly in recent decades, establishing an MTB is crucial to fulfilling the requirements of multimodal treatments. Because of this, the MTB approach for colorectal cancer has been widely accepted and is recommended worldwide as the standard of care in current practice.^12-14^ The purpose of this study was to describe the profile of patients discussed in the Tikur Anbessa MTB and the potential benefits brought by multidisciplinary care.

## METHODS

### Study Setting

Tikur Anbessa Hospital is the largest referral and teaching hospital in Ethiopia. Until now, it has been the only center in which patients with cancer are treated at a tertiary level and where access to chemotherapy and radiotherapy are available. The Department of Clinical Oncology has two radiotherapy machines (cobalt-60), 36 beds in the impatient chemotherapy ward, and 12 outpatient chemotherapy beds. Six clinical oncologists serve the department. Patients who need chemotherapy and radiotherapy are referred to this hospital from the whole country. The Department of Surgery has two colorectal surgeons. Currently, the Department of Radiology is equipped with two computed tomography scans and one magnetic resonance imaging unit. The Department of Pathology provides histologic results mainly on the basis of microscopic morphology. Immunohistochemical tests are performed intermittently on patients with malignant disease.

### Tikur Anbessa Colorectal Cancers MTB

The Tikur Anbessa Hospital colorectal cancers MTB, established on May 2014, is the first tumor board in Ethiopia. It is composed of surgeons specializing in oncologic interventions (colorectal surgeons and a hepatobiliary surgeon), clinical oncologists, and radiologists. Residents from various specialties also attend the tumor board as a part of their education. The Tikur Anbessa Hospital Colorectal Cancers MTB conducts a meeting once a week, every Friday.

In May 2016, the Tikur Anbessa Hospital colorectal cancers MTB was reorganized to facilitate the board’s effectiveness. The Department of Clinical Oncology assigns the coordinator for all activities of the tumor board. The basic role of the coordinator is to accept patient cases selected from each department for tumor board discussion and to send, via e-mail, the medical summary of each of the selected patient cases to all attending physicians 1 day before the meeting to keep them informed. In addition, presenting the patient cases, moderating the meeting, and recording the discussion and decisions after discussion are the responsibilities of the coordinator. After each session, the decisions made at the meeting are sent to all attending physicians by e-mail, and the printout is filed in each department for future reference. The patients are managed according to the agreed-on consensus.

### Study Design

The study involved assessment of all patients presented to the Tikur Anbessa Hospital colorectal cancers MTB between March 2016 and November 2017. We retrospectively evaluated all patients presented to the tumor board from the documents containing the medical summary of each selected patient and the decisions made. Documentation contains data on cancer type, cancer histology, initial staging data before MTB presentation, previous therapeutic procedures, and tumor board recommendation. The staging was made according the TNM staging system of the American Joint Committee on Cancer, 7th edition.^15^ The confidentiality of the patient and the attending physician was maintained during data collection and analysis. The descriptive analysis was performed using SPSS version 20 software.

The study was conducted according to Addis Ababa University ethical guidelines, and approval was received from the Department of Clinical Oncology Research Committee. The study was conducted without individual informed consent, because it relied on retrospective data collected as part of routine patient care and the data were anonymous.

## RESULTS

One hundred fifty-four patients were presented between March 2016 and November 2017 to Tikur Anbessa Hospital colorectal cancers MTB. Of these, 147 patients (95%) had colorectal or anal cancers and seven (5%) had cancers of the hepatobiliary system.

**FIG 1:**
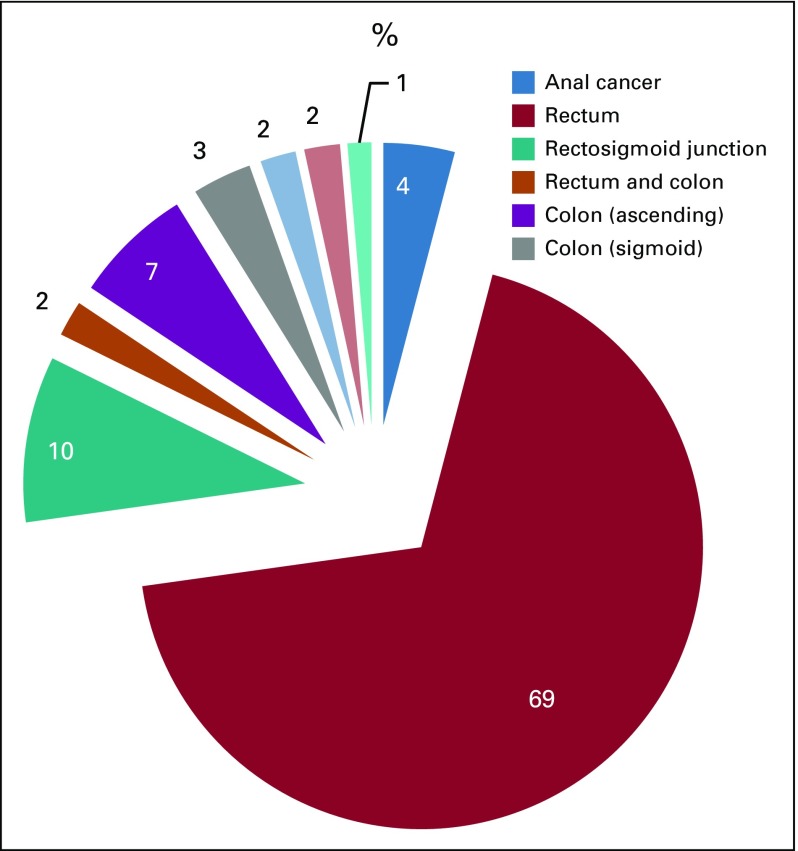
Distribution of patient cases by anatomic site of the primary cancer.

Of the 147 patients with colorectal cancer, 96 (65%) were men. The median age at presentation was 46 years (range, 17-78 years). The anatomic site of primary cancer was predominantly rectal (n = 101; 69%), followed by colon (n = 24; 16%), rectosigmoid junction cancer (n = 13; 9%), anal (n = 6; 4%), and synchronous colon and rectal (n = 3; 2%; Fig 1).

**FIG 2:**
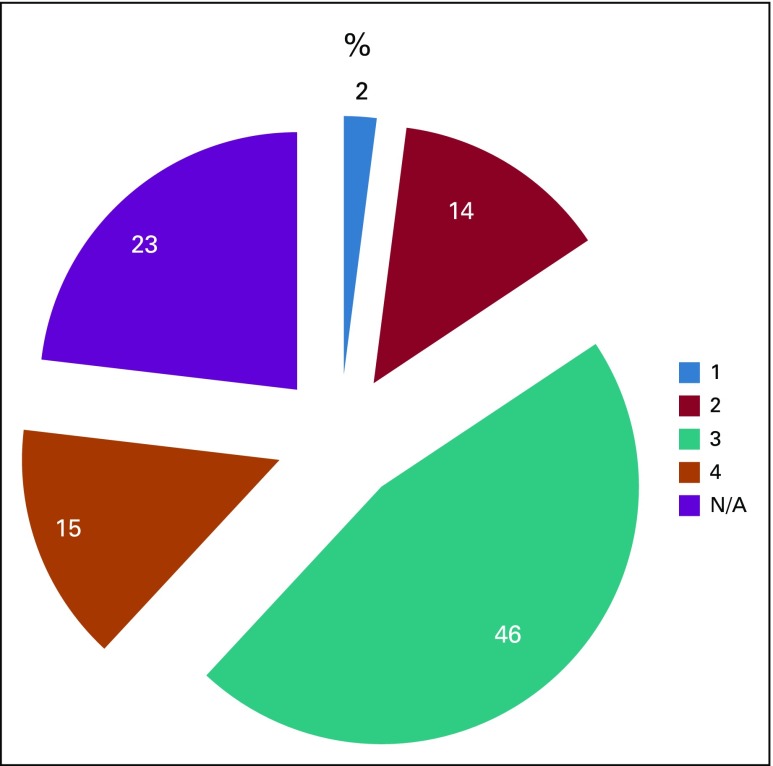
Distribution of patient cases by American Joint Committee on Cancer–stage of disease at the time of tumor board presentation. N/A, not available.

Of the patients with colorectal and anal cancer, 68 (45%) were diagnosed with stage III, 20 (14%) with stage II, 22 (15%) with stage IV, and three (2%) with stage I cancer on presentation to the tumor board. The rest (n = 34; 23%) had incomplete information for staging (Fig 2).

The histologic diagnosis of all patients with colorectal cancer, including anal cancer, was mainly adenocarcinoma (n = 136; 93%). There were six patient cases of squamous cell carcinoma (4%), and the other histologies included carcinoid tumor, GI stromal tumor, melanoma, myxiod fibrosarcoma, and liposacroma (one patient case each [1%]; Table 1).

**TABLE 1:**
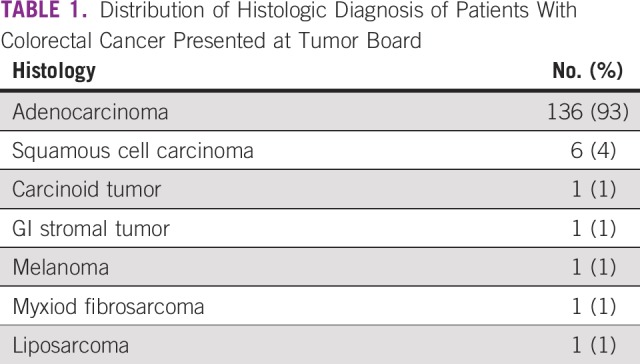
Distribution of Histologic Diagnosis of Patients With Colorectal Cancer Presented at Tumor Board

Of the 147 patient cases, physicians from the Department of Clinical Oncology presented 127 patients (86%) for MTB discussion, whereas physicians from the Department of Surgery presented 20 patients (14%). All physicians participated in patient case discussions and in the making of management plans.

Of the 147 patients, 66 (45%) had received oncologic treatment before their presentation to the MDT; 81 (55%) had not received any kind of oncologic treatment before and they presented as new patient cases.

### Characteristics of Patients Who Received Oncologic Treatment Before Presentation to Tumor Board

Of the 66 patients who had received treatment before presentation to the MTB, the anatomic location of the tumor was 37 (56%) rectal, 18 (27%) colon, nine (14%) rectosigmoid, and two (3%) anal (Table 2). The prior oncologic treatment consisted of radical operation for 58 patients (88%), neoadjuvant chemotherapy for six (9%; all patient cases of rectal cancer), and tumor resection for two (3%). The majority of patient cases (n = 32; 48%) did not have complete information for proper staging, mainly because there was incomplete preoperative clinical stage information on the referral paper. In addition, none had postoperation nodal status information and of these, seven did not have postoperation pathologic tumor stage information either. Thirty-four patients (52%) had adequate information for staging; of these, 15 patients had stage III, 11 patients had stage IV, seven had stage II, and one had stage I cancer at the time of presentation.

**TABLE 2:**
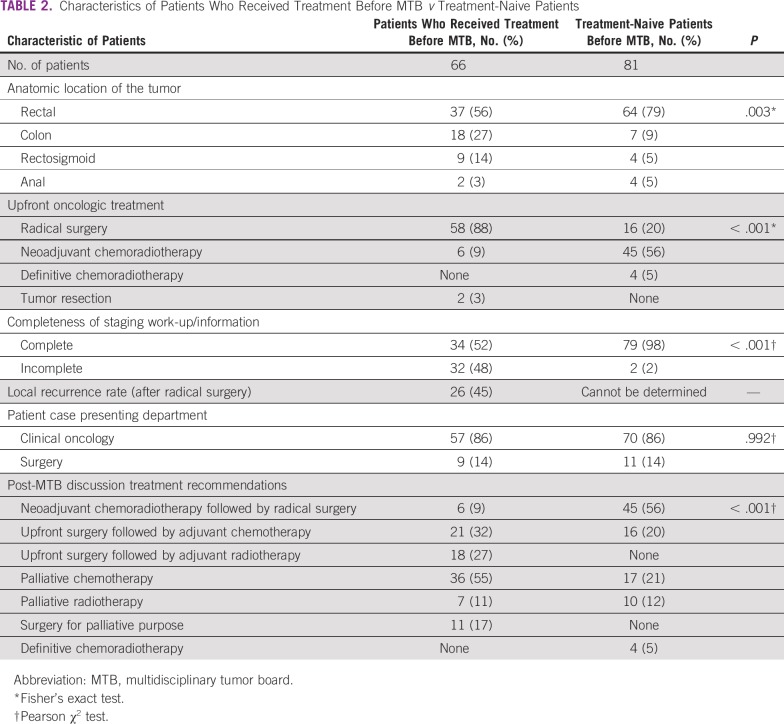
Characteristics of Patients Who Received Treatment Before MTB *v* Treatment-Naive Patients

Among 58 patients for whom radical surgery was performed, 31 (64%) surgeries were rectal, 18 (31%) colonic, and nine (16%) rectosigmoid. Of these, 34 patients (58%) underwent surgery on an elective basis, five (9%) underwent surgery on an emergency basis, and all were colon cancers with obstruction. For the remaining 19 patients (33%), the mode of surgical presentation was not documented. Thirty-one patients with rectal cancer had undergone upfront surgery without neo-adjuvant chemoradiotherapy. The most common operation performed for those with rectal cancer was abdominoperineal resection in 22 patients (71%) and lower anterior resection in eight (26%), and in one patient case, the cancer was known to be inoperable intraoperatively. For nine patients with rectosigmoid junction tumor, lower anterior resection was performed for seven patients and hemicolectomy for two patients. Hemicolectomy was the procedure performed in all 18 patients who underwent surgery for colon cancer. From the pathology reports of the patients who underwent surgery, 11 (19%) had involved surgical margin status and 11 (19%) were free of tumor surgical margin status. However, 36 patients had no documentation of surgical margin status. Among patients who underwent radical surgery before MDT, local recurrence was the reason for presentation in 26 (45%).

Of all 66 patients who received prior oncologic therapy, 57 (86%) were presented to the MTB by the Department of Clinical Oncology and nine (14%) by the Department of Surgery. After discussion, the tumor board decision was palliative chemotherapy for 36 patients (55%), adjuvant chemotherapy for 21 (32%), adjuvant radiotherapy for 18 (27%), surgery for palliative purpose for 11 (17%), and palliative radiotherapy for seven (11%). For a number of patients, various combinations of the previously mentioned individual treatment plans were selected.

### Characteristics of Patients Who Did Not Receive Oncologic Treatment Before Presentation to Tumor Board

Eighty-one patients were presented to the tumor board as new patient cases before any oncologic therapy had been received (Table 2). Of these, 64 patients (79%) had rectal cancer, seven (9%) had colon cancer, four (5%) had anal and rectosigmoid tumors, and two (2%) had synchronous colon and rectal tumors. Of all the patients, 79 (98%) were properly staged as per the American Joint Committee on Cancer, 7th edition staging manual and two did not have proper staging. Of these 79 patients, 52 (64%) had stage III, 13 (16%) had stage II, 12 (15%) had stage IV, and two (2%) stage I disease.

After discussion in the MTB, the decisions were neoadjuvant chemoradiotherapy followed by surgery for 45 patients (56%); palliative chemotherapy for 17 patients (21%) and of these, palliative radiotherapy for 10 patients (12%) as well; upfront radical surgery followed by adjuvant chemotherapy or radiotherapy for 16 patients (20%); and definitive chemoradiotherapy for four patients (5%).

Of all 81 patients who had not received prior oncologic therapy, 70 (86%) were presented to the MTB by the Department of Clinical Oncology; the Department of Surgery presented 11 patients (14%).

## DISCUSSION

In this study, we have summarized the experience of the first MTB in Ethiopia during its first year of establishment. We noted from our study that two thirds of patient cases presented to the tumor board were patients with rectal cancer and one third were patients with colonic cancer. The relative number of patient cases of colon cancer versus rectal cancer was found to be opposite to those presented in Western countries.^16^ This is possibly because the current management recommendation for rectal cancer in many patient cases requires combination radiotherapy and chemotherapy,^13^ which is only available in a specialized hospital, and this leads to more common referral to the MTB. Nevertheless, in Ethiopia, specific oncologic therapy including chemotherapy is only possible within specialized centers. It may be hypothesized that a majority of patients with colon cancer are not presented to tumor boards or do not receive necessary adjuvant chemotherapy. Conversely, previous studies of pathologic biopsies of colorectal cancers in Ethiopia reported that two thirds of the patient cases were rectal cancer and one third were colonic cancer.^17^ This is consistent with our findings. Other studies from surgical departments have also shown that rectal cancer comprises one half of all of surgeries for colorectal cancer, which is still a higher percentage than that reported by Western countries.^18^ The other finding of our study, which is different from the findings of Western studies, was the younger age at presentation (ie, a median age of 46 years). Other studies on colorectal cancer in Ethiopia have reported a similar median age at presentation.^17,18^ However, additional study is mandatory to characterize the current epidemiology of colorectal cancer in Ethiopia.

Evidence indicates that decisions made by MTBs are more likely to conform to evidence-based guidelines than are those made by individual clinicians.^19-22^ We also found that the management plan for patient cases presented to tumor boards from the beginning, as new patients, conformed more to an evidence-based approach, compared with those patients who had received treatment before MTB presentation. For example, with regard to proper staging and the provision of neoadjuvant chemoradiotherapy for patients with rectal cancer, those patients who were presented early to the MTB had better treatment plans, as per current guidelines. More than one half of patients (52%) who underwent surgery before the tumor board had inadequate staging information compared with those patient cases presented initially, where 98% had complete clinical staging. It is obvious that accurate tumor staging for colorectal cancer, as well as other types of cancer, is critical to define appropriate management, to facilitate communication between physicians, to provide a basis for stratification and analysis of treatment results in studies, and to provide some prognostic information for patients and their families.^12,13,23^ Therefore, properly staged patients will have a better chance of a proper treatment plan, which will result in better patient selection and better oncologic outcome.^24^

Among patients with rectal cancer who were presented as new patients to the tumor board, most (70%) planned to have neoadjuvant chemoradiotherapy followed by surgery. In contrast, in previously treated patients, only 16% were able to receive neoadjuvant chemoradiotherapy, and others underwent surgery up front. Because most of these patients were at a locally advanced stage, preoperative chemoradiotherapy was preferred for a better oncologic outcome, especially in terms of the local failure control rate.^13,25-29^ This can be illustrated by our finding that the postsurgical local recurrence rate is 45% among those who underwent surgery before tumor board presentation. However, although we cannot compare these patients with the patients managed after tumor board discussion right from the beginning, it is an obviously unacceptably higher recurrence rate than those of older Western studies that reported a local recurrence rate of < 13%.^30^ It is important to note that the higher recurrence rate is not merely a result of the absence of an MTB presentation. It could be caused by poor patient selection, because one half of patient cases with recurrence had no proper staging. Other possible reasons could be poor surgical technique or a long waiting time for chemoradiotherapy because of the limited access to radiotherapy and chemotherapy in Ethiopia.^31^ However, the findings show that patients treated after MTB discussion from the beginning had a better management plan as per guidelines.^13,26^

This study shows that the colorectal cancers MTB in Tikur Anbessa Hospital is used as a forum for group consultations and multidisciplinary patient management. We believe this is a positive trend toward better and multidisciplinary care of patients with cancer in the hospital. Although clinical oncologists presented the majority of the patient cases, surgeons and radiologists were essential in discussions and final management plans. All members of the tumor board were important contributors to the success of the tumor board by virtue of case preparation, presentation, discussion, and the making of management plans. The tumor board discussion also became a good platform for teaching residents and junior doctors. It also creates good interdepartmental relationships, and it has become a source for joint research and clinical trials. However, the low rate of referral from the surgical department, poor adherence to guidelines for those who underwent surgery before presentation to the MTB, and poor documentation of findings are challenges that must be addressed. In addition, the lack of participation by pathologists is one of the deficiencies of this tumor board. Pathologists may not have participated because of other overlapping obligations, including conducting procedures or teaching. However, we recommend that in addition to the already existing physicians, pathologists and other specialists such as gastroenterologists and supporting nurse staff participate in discussions and decision making, to expand multidisciplinary care to all patients with colorectal cancer and to improve the quality of service. Proper training regarding the current management guidelines for surgeons and other relevant stakeholders and placing mechanisms to strengthen the referral system between departments must be emphasized.

We believe the multidisciplinary cancer management started by the Tikur Anbessa Hospital colorectal cancers MTB is highly beneficial to the hospital and its patients. It should be expanded to other types of cancer as well. It is also important for other hospitals in Ethiopia to learn from the practice of this tumor board and to establish multidisciplinary cancer management to improve the quality of service for their patients.

Because the patient cases presented to the Tikur Anbessa Hospital colorectal cancers MTB for discussion were selected patients, the clinicopathologic features we found during our analysis may not represent the actual characteristics of patients with colorectal cancer in this country. The other limitation of our study is that the information included concerned mainly the characteristics of patients at their first tumor board presentation, not the outcome after treatment.

The MTB enhanced integrated cancer care and resulted in better staging accuracy and management. Decisions made by the MTB are more likely to conform to evidence-based guidelines than are those made by individual clinicians. Therefore, early referral of patients to the MTB before any treatment must be practiced by responsible physicians to result in better oncologic outcomes. Finally, other hospitals in Ethiopia should take a lesson from the Tikur Anbessa Hospital colorectal cancers MTB and adopt multidisciplinary cancer management for all patients with cancer.

## Data Availability

The following represents disclosure information provided by authors of this manuscript. All relationships are considered compensated unless otherwise noted. Relationships are self-held unless noted. I = Immediate Family Member, Inst = My Institution. Relationships may not relate to the subject matter of this manuscript. For more information about ASCO's conflict of interest policy, please refer to www.asco.org/rwc or ascopubs.org/jgo/site/misc/authors.html. Open Payments is a public database containing information reported by companies about payments made to US-licensed physicians (Open Payments). **Employment:** niAnalytics GmbH **Leadership:** niAnalytics GmbH **Stock and Other Ownership Interests:** niAnalytics GmbH No other potential conflicts of interest were reported.
